# Strangulated Morgagni's Hernia: A Rare Diagnosis and Management

**DOI:** 10.1155/2016/2621383

**Published:** 2016-11-07

**Authors:** Malav Modi, Amit Kumar Dey, Ajay Mate, Samir Rege

**Affiliations:** Department of Surgery, Seth G.S. Medical College and KEM Hospital, Mumbai, India

## Abstract

Morgagni hernia is a rare type of congenital diaphragmatic hernia. It accounts for only 3% of all diaphragmatic hernias. The defect is small and hernia being asymptomatic in the majority presents late in adulthood. Obstruction or incarceration in Morgagni hernia is uncommon. We report a rare occurrence of strangulated Morgagni hernia. A 40-year-old gentleman presented to our emergency department with features of intestinal obstruction. Computed tomography of the chest and abdomen showed a strangulated right Morgagni hernia. An exploratory laparotomy was performed with resection of the ischemic bowel segment with anastomosis and a primary repair of the diaphragmatic defect. Postoperative recovery was uneventful and asymptomatic at follow-up.

## 1. Introduction

Morgagni's hernia is the herniation of the intra-abdominal organs through a congenital defect in the diaphragm immediately behind the sternum [1]. Its cases are very rare and make up 2-3% of all cases among the four types of congenital diaphragmatic hernias [2]. Though some are symptomatic, many remain asymptomatic for a long time and are discovered on X-ray incidentally [3]. Strangulated Morgagni's hernia is very uncommon and only five such cases are reported in literature [4–6]. This paper is written with the objective of reporting a new case of strangulated Morgagni's hernia along with its morphology and treatment in detail.

## 2. Case Presentation

**Figure 1:**
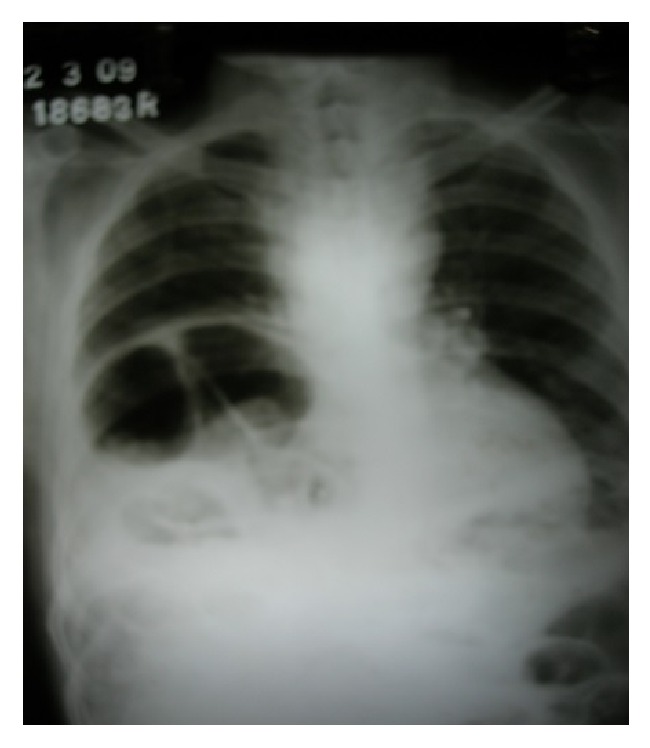
Chest X-ray showing an elevated right hemidiaphragm.

**Figure 2:**
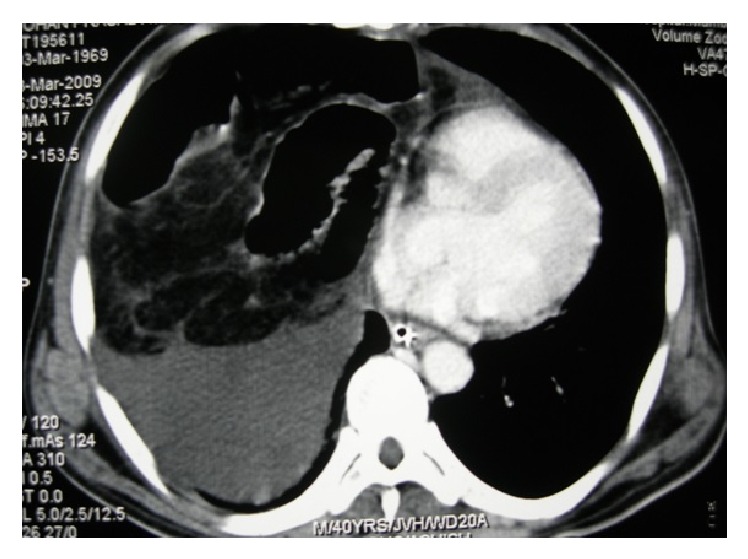
Preoperative CT scan showing thickened small bowel loops in the right hemithorax with air specks in the bowel wall suggesting a strangulated diaphragmatic hernia.

**Figure 3:**
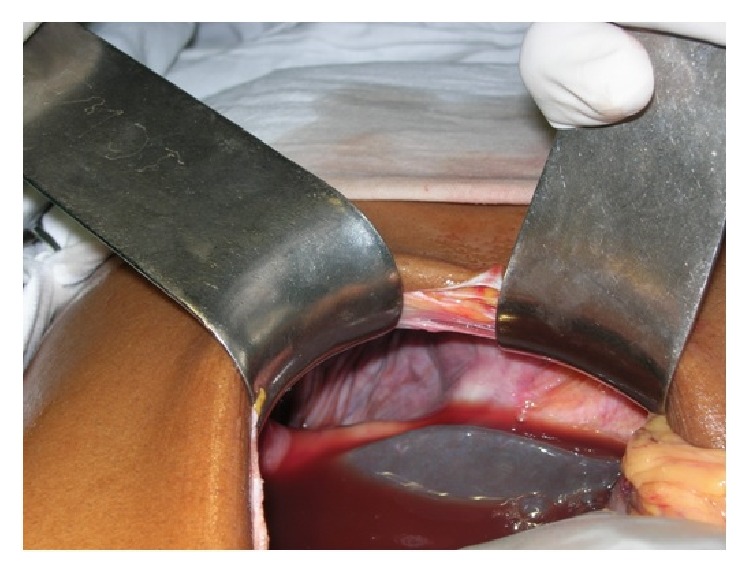
Intraoperative photo showing the inflammatory haemorrhagic fluid around the diaphragmatic defect.

**Figure 4:**
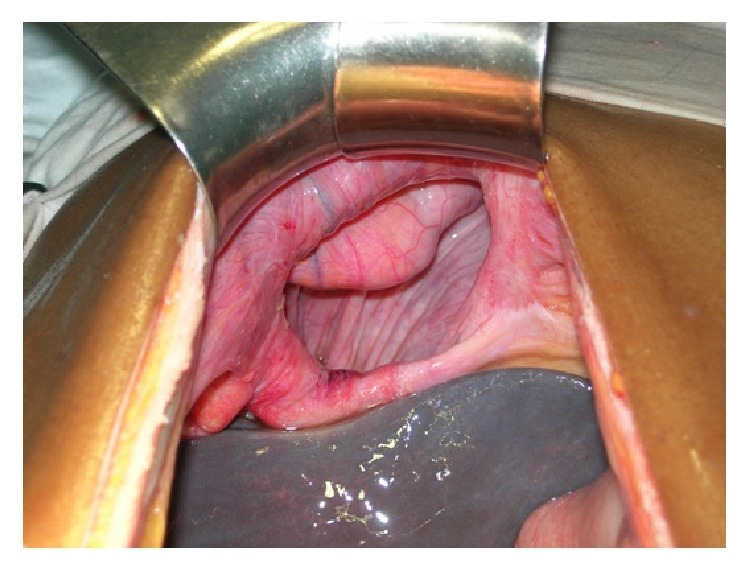
Intraoperative photo showing the right sided Morgagni's hernial defect.

A 40-year-old male patient presented to our emergency department with acute severe pain and distension of abdomen. Severe abdominal pain was associated with absolute constipation and multiple episodes of vomiting that started 2 days ago. He also complained of increasing breathlessness since then. Patient was a chronic alcoholic and an occasional smoker. There was no prior history of similar complaints in the past. On examination, patient was normotensive with persistent tachycardia and tachypnoea. Abdomen was distended, tender, guarded with no signs of peristalsis on auscultation. Air entry was reduced on right lower zone of chest on auscultation. Chest radiograph showed an elevated right hemidiaphragm (Figure 1). Scout film of the abdomen revealed multiple air fluid levels with elevated right hemidiaphragm. Ultrasonography of the abdomen was indicative of dilated small bowel loops with ascites with right pleural effusion. Computed tomogram (CT) of the chest and abdomen performed was suggestive of herniation of jejunoileal loops through a right diaphragmatic defect (Morgagni's hernia) with ascites and right pleural effusion (Figure 2). After initial resuscitation, patient was explored through a midline laparotomy incision. There was a defect in the diaphragm on the right side anteriorly 10 × 6 cm in diameter between sternal and costal attachments of diaphragm with herniation of jejunoileal loops (Figures 3 and 4). Jejunoileal loops of around 50 cm were gangrenous and aperistaltic with poor turgor. The sac also contained about 400 mL of dark haemorrhagic fluid. The right lung was hypoplastic. Hernial contents were reduced and the ischemic segment of bowel was resected followed by an anastomosis. Peritoneal sac was dissected. Right intercostal drain was placed and pleura was sutured. The diaphragmatic defect was closed with an Ethibond 2-0. Postoperatively, patient was on ventilator support for 3 days. He was gradually weaned and extubated. Intercostal drain was removed after complete lung expansion. Gradually, oral feeds were started which patient tolerated well. The patient made an uneventful recovery and is now asymptomatic at one-year follow-up.

## 3. Discussion

Failure of fusion of the fibrotendinous elements of diaphragm, that is, sternal and costal attachments, leaves behind a muscle-free area known as the costosternal trigone or the space of Larrey or Morgagni's foramen through which the hernia occurs eventually [1]. Because of extensive pericardial attachment on the left which does not allow herniation, the hernia is more common on the right side and is situated anteriorly [7]. Almost 90% of Morgagni's hernias are reported to be on the right side, with 2% located on the left and 8% bilateral [8]. The sac lies between the pericardium and the right pleura. Comer and Clagett reported a series of 1135 cases of diaphragmatic hernia at Mayo's clinic, of which only 50 were of Morgagni's type with only one obstructed case [2]. Usually, the hernia sac contains the transverse colon followed by stomach, omentum, and small intestine but occasionally the liver may also protrude into the sac [9]. Diagnosis of Morgagni's hernias is usually late because patients can be asymptomatic or present with vague respiratory (cough, expectoration, and dyspnea) or gastrointestinal (nausea, vomiting, subcostal pain, pain after food, or rarely acute intestinal obstruction) symptoms and signs [10]. Diagnosis is usually done by conventional radiography with occasionally missed cases. Computed tomography scan can be useful in diagnosing the contents of the hernia sac and is noninvasive and accurate. Magnetic resonance imaging can distinguish Morgagni's hernia from other mediastinal masses and is noninvasive too [8]. Pleuropericardial cyst, lipoma, intrathoracic tumours, and eventration of diaphragm should be differentiated from Morgagni's hernia while making a diagnosis [9]. Barium studies could be useful in supplementary investigation. In our case, diagnosis was made by physical examination and plain chest radiogram and confirmed by computed tomography. To avoid the risk of strangulation, some recommend repair even in asymptomatic patients [5] whereas some advocate conservative approach because Morgagni's hernia remains asymptomatic for a long time [11]. A subxiphoid preperitoneal approach has the benefit of a small incision [9]. In cases of certain diagnosis, abdominal approach (open or laparoscopic) is preferred over thoracic approach for surgery because of easier reduction of the hernia and because abdominal viscera within the hernia can be easily pulled down to their normal location [8]. In cases of unclear diagnosis, the recent trend is towards laparoscopy which has low morbidity [6]. Laparoscopy can be therapeutic, as well as diagnostic [12]. In our case, open abdominal approach was preferred because of ischemic bowel and general condition of patient. In conclusion, Morgagni's hernia is a rare type of congenital diaphragmatic hernia and is more common on right side.

## 4. Conclusion

Most of the patients are asymptomatic and present late usually with complications. Diagnosis in suspected case can be confirmed by computed tomography scan. Surgery by abdominal approach (open or laparoscopic) is management of choice.
